# Comparison of the surgical outcomes of minimally invasive and open surgery for octogenarian and older compared to younger gastric cancer patients: a retrospective cohort study

**DOI:** 10.1186/s12893-017-0265-3

**Published:** 2017-06-12

**Authors:** Chien-An Liu, Kuo-Hung Huang, Ming-Huang Chen, Su-Shun Lo, Anna Fen-Yau Li, Chew-Wun Wu, Yi-Ming Shyr, Wen-Liang Fang

**Affiliations:** 1 Department of Radiology, Taipei Veterans General Hospital, Taipei City, Taiwan; 2 Division of General Surgery, Department of Surgery, Taipei Veterans General Hospital, No. 201, Sec. 2, Shipai Rd., Beitou District, Taipei City, 11217 Taiwan; 3 Division of Medical Oncology, Department of Oncology, Taipei Veterans General Hospital, Taipei City, Taiwan; 4 Department of Pathology, Taipei Veterans General Hospital, Taipei City, Taiwan; 5 School of Medicine, National Yang-Ming University, Taipei City, Taiwan; 6 Institute of Clinical Medicine, School of Medicine, National Yang-Ming University, Taipei City, Taiwan; 7 National Yang-Ming University Hospital, Yilan City, Taiwan

**Keywords:** Minimally invasive surgery, Open surgery, Octogenarian and older, Gastric cancer

## Abstract

**Background:**

As life expectancy continues to increase around the world, the use of minimally invasive surgery (MIS) could be beneficial for octogenarian and older gastric cancer patients.

**Methods:**

A total of 359 gastric cancer patients who underwent curative surgery between March 2011 and March 2015 were enrolled; 80 of these patients (22.2%) were octogenarians and older. Surgical approaches included MIS (50 laparoscopic and 65 robotic) and open surgery (*n* = 244). Surgical outcomes of MIS and open surgery in octogenarian and older patients were compared with younger patients.

**Results:**

Among octogenarian and older patients, relative to open surgery (*n* = 53), MIS (*n* = 27) was associated with less operative blood loss, a shorter postoperative hospital stay and similar rates of surgical complications and mortality. For MIS (*n* = 115), octogenarian and older patients exhibited similar postoperative outcomes to those of younger patients. For open surgery (*n* = 244), relative to younger patients, octogenarian and older patients experienced longer postoperative hospital stays, a higher rate of wound infection and a higher incidence of pneumonia.

**Conclusions:**

MIS for gastric cancer is beneficial and can be performed safely in octogenarian and older patients.

## Background

As life expectancy continues to increase around the world, the proportion of octogenarian and older patients who undergo gastrectomies for gastric cancer is also increasing. Advanced age is frequently associated with significant comorbidity and a limited functional reserve; given these characteristics, octogenarian and older patients generally exhibit higher rates of complications and longer hospital stays than younger patients.

Advantages of minimally invasive surgery (MIS) relative to open surgery include less wound pain, earlier functional recovery and shorter hospital stay [1–3]. Surgical treatment is the only known approach for curing gastric cancer. The advantages of MIS might cause octogenarian and older patients with high risks of operative morbidity and mortality to become more willing to receive surgery instead of strictly conservative treatment.

To date, only one study has compared MIS with open surgery for octogenarian and older gastric cancer patients [4]. In that series, relative to open surgery, MIS was associated with significantly less blood loss, lower analgesic consumption, faster time to first flatus and a soft diet, and a shorter postoperative hospital stay. Therefore, MIS for gastric cancer may be performed safely in octogenarian and older patients; in this context, MIS continues to exhibit the advantages associated with minimal invasiveness.

The aim of this study was to compare the outcomes of MIS and open surgery for octogenarian and older compared to younger gastric cancer patients, in terms of mortality, complication rate, blood loss and hospital stay.

## Methods

Between March 2011 and March 2015, a total of 359 gastric cancer patients were enrolled in this study. All consecutive patients were treated for gastric cancer in our institution. The current study was approved by the institutional review board of Taipei Veterans General Hospital. Pathological stages were determined in accordance with the 7th edition of the classification guidelines issued by the American Joint Committee on Cancer [5].

All the operations were performed by surgeons who specialized in gastric cancer. We retrospectively reviewed our gastric cancer database that was prospectively collected and regularly updated using a computer. Prior to surgery, all patients underwent chest radiography, abdominal sonography, or a CT scan for tumor staging. The patients were evaluated on the basis of their gender, age, tumor size, tumor location, operative methods, pathological tumor and lymph node stage, lymphovascular invasion, comorbidities, stromal reaction type and gross appearance.

Most of the patients received continuous intravenous or epidural injection of mixed analgesics for 3–4 days after surgery. Water was started on postoperative day 3 or day 4, and a soft diet was started on postoperative day 5 to day 7. The patient was discharged if no complication occurred.

### Indication for open and minimally invasive (laparoscopic or robotic) gastrectomy

At our hospital, the indications for laparoscopic or robotic gastrectomy are the same, which is gastric cancer at a less advanced clinical stage than T3N1M0. Patients who had a history of gastric surgery or were referred to gastrointestinal endoscopists for endoscopic mucosal resection or endoscopic submucosal dissection were excluded from this study.

Compared with laparoscopic or robotic gastrectomy, the indication of open surgery is not limited to the clinical stage. No matter what stage the cancer is at, patients who are diagnosed as gastric cancer can choose open surgery of their own will.

The surgeons comprehensively explained the advantages and disadvantages of the three possible surgical approaches to each patient prior to surgery. After receiving this explanation, patients decided which surgical approach would be utilized and provided written informed consent.

A total or distal subtotal gastrectomy is performed, depending on the distance between the cardia and the tumor. A margin of 3 cm is needed for superficial and well defined tumors; a margin of 5 cm is needed for advanced or poorly defined tumors. A subtotal gastrectomy is the standard procedure for distal gastric cancer, whereas a total gastrectomy is the more common procedure for proximal gastric cancer.

All patients were subjected to gastrectomy with at least D1 + α (perigastric lymph nodes + No. 7 lymph nodes) or D1 + β (perigastric lymph nodes + Nos. 7, 8, 9 lymph nodes) for early gastric cancer and D2 lymphadenectomy for advanced gastric cancer.

### Statistical analysis

Statistical analysis was conducted using IBM SPSS Statistics V22.0. The chi-square test was used to compare categorical variables between groups, while the independent Student’s t-test was used to evaluate the difference in continuous variables between the minimally invasive surgery group and the open surgery group. The one-way analysis of variance (ANOVA) was used to determine whether there are any statistically significant differences in the continuous variables between the three different age groups. *P* values less than 0.05 were considered to be statistically significant.

## Results

A total of 359 gastric cancer patients who received curative resection in our institution were enrolled in this study. In particular, 80 patients (22.3%) were octogenarian or older, 167 patients (46.5%) were 60–79 years of age, and 112 patients (31.2%) were younger than 60 years of age. Among the 359 patients, 115 patients (32%) underwent MIS and 244 patients (68%) underwent open surgery.

Surgical mortality occurred in two cases, both of which involved open surgery. In particular, an 81-year-old male patient who underwent total gastrectomy died of aspiration pneumonia. In addition, a 78-year old female patient who received total gastrectomy experienced esophagojejunostomy leakage and peritonitis. She underwent an exploratory laparotomy, multiple drainage procedures and esophageal stent implantation; but eventually died of sepsis and multiple organ failure.

### Octogenarian and older patients

As indicated in Table 1, among the 80 octogenarian and older patients, a comparison of MIS and open surgery indicated that MIS was associated with higher BMI (24.4 ± 3.1 vs. 22.7 ± 3.5, *P* = 0.032), more subtotal gastrectomy (96.3% vs. 73.6%, *P* = 0.015), less advanced pathological T category (*P* = 0.017), less operative blood loss (63.7 ± 59.2 mL vs. 372.3 ± 340.4 mL, *P* < 0.001) and shorter hospital stay (10.7 ± 8.6 vs. 15.4 ± 9.7 days, *P* = 0.036). The surgical complication rates (22.2% vs. 11.3%, *P* = 0.207) and surgical mortality rates (0% vs 1.9%, *P* = 1.000) were not significantly different between MIS and open surgery in octogenarian and older patients.

**Table 1:** Comparison of the clinicopathological differences between minimally invasive gastrectomy and open gastrectomy for octogenarian and older gastric cancer patients

	Minimally invasive gastrectomy *n* = 27	Open gastrectomy *n* = 53	*P* value
Age (years)	84.3 ± 3.3	84.1 ± 3.2	0.831
Gender (M/F)	19/8	41/12	0.587
Tumor size (cm)	3.9 ± 2.3	4.3 ± 2.5	0.478
BMI (kg/m^2^)	24.4 ± 3.1	22.7 ± 3.5	0.032
Resection extent			0.015
Subtotal/total gastrectomy	26/1	39/14	
Reconstruction method			0.345
Billroth-I	6 (22.2)	7 (13.2)	
Roux-en-Y or uncut R-Y	21 (77.8)	46 (86.8)	
Extent of lymphadenectomy			
<D2/D2	2/25	6/47	0.710
Retrieved LN number	26.0 ± 10.1	28.4 ± 13.4	0.428
Pathological T category			0.017
T1/T2/T3/T4	17/3/6/1	17/12/17/7	
Pathological N category			0.058
N0/N1/N2/N3	20/2/4/1	29/4/12/8	
Pathological TNM stage			0.051
I/II/III	18/6/3	22/13/18	
Number of comorbidities			0.363
0	5 (18.5)	9 (17)	
1	6 (22.2)	22 (41.5)	
≧2	16 (59.3)	22 (41.5)	
Operative outcomes			
Operative time (min)	311.5 ± 106.6	313.2 ± 101.9	0.945
Operative blood loss (mL)	63.7 ± 59.2	372.3 ± 340.4	<0.001
Postoperative hospital stay (day)	10.7 ± 8.6	15.4 ± 9.7	0.036
Surgical complications	6 (22.2)	6 (11.3)	0.207
Anastomosis leakage	1 (3.7)	1 (1.9)	1.000
Anastomosis stenosis	1 (3.7)	0	0.337
Delayed gastric emptying	4 (14.8)	2 (3.8)	0.172
Intestinal obstruction	1 (3.7)	0	0.337
Pneumonia	0	2 (3.8)	0.547
Surgical Mortality	0	1 (1.9)	1.000

BMI: body mass index; LN: lymph node; comorbidities including cardiovascular disease, cerebrovascular accident, endocrine disease, pulmonary disease, liver cirrhosis, benign prostate hyperplasia, etc.

Some patients had more than one complication

Data were presented as mean ± SD or n (%)

### Minimally invasive surgery

As indicated in Table 2, MIS was performed on 115 patients, including 62 females (53.9%) and 53 males (46.1%). The median age of these patients was 68 years (range, 35–91 years). Patients were classified into three groups according to their ages. Group 1 included 40 patients (34.8%) younger than 60 years of age (18 males and 22 females), with a median age of 51 years (range, 35–59 years). Group 2 included 48 patients (41.7%) of 65 to 79 years of age (25 males and 23 females), with a median age of 70.5 years (range, 60–79 years). Group 3 included 27 patients (24.5%) of at least 80 years of age (19 males and 8 females), with a median age of 84 years (range, 80–91 years).

**Table 2:** Comparison of the clinicopathological differences and operative outcomes of minimally invasive gastrectomy according to age

	<60 yr	60–79 yr	≧80 yr	*P* value
	*n* = 40	*n* = 48	*n* = 27	
Gender (M/F)	18/22	25/23	19/8	0.117
Tumor size (cm)	3.5 ± 1.5	3.5 ± 1.6	3.9 ± 2.3	0.574
BMI (kg/m^2^)	23.7 ± 4.1	23.8 ± 3.2	24.4 ± 3.1	0.713
Resection extent				0.005
Subtotal/total gastrectomy	30/10	45/3	26/1	
Reconstruction method				0.012
Billroth-I	11 (27.5)	25 (52.1)	6 (22.2)	
Roux-en-Y or uncut R-Y	29 (72.5)	23 (47.9)	21 (77.8)	
Extent of lymphadenectomy				
<D2/D2	3/36	1/47	2/25	0.838
Retrieved LN number	35.0 ± 10.4	29.7 ± 12.0	26.0 ± 10.1	0.005
Pathological T category				0.820
T1/T2/T3/T4	25/5/6/4	30/4/12/2	17/3/6/1	
Pathological N category				0.271
N0/N1/N2/N3	26/4/4/6	31/7/8/2	20/2/4/1	
Pathological TNM stage				0.390
I/II/III	22/12/6	28/14/6	18/6/3	
Number of comorbidities				<0.001
0	28 (70)	18 (37.5)	5 (18.5)	
1	7 (17.5)	15 (31.3)	6 (22.2)	
≧2	5 (12.5)	15 (31.3)	16 (59.3)	
Operative outcomes				
Operative time (min)	344.9 ± 142.9	270.3 ± 109.9	311.5 ± 106.6	0.019
Operative blood loss (mL)	59.8 ± 59.9	71.3 ± 74.1	63.7 ± 59.2	0.710
Postoperative hospital stay (day)	9.5 ± 6.4	12.2 ± 14.0	10.7 ± 8.6	0.499
Surgical complications	3 (7.5)	8 (16.7)	6 (22.2)	0.088
Anastomosis leakage	0	1 (2.1)	1 (3.7)	0.249
Anastomosis stenosis	1 (2.5)	1 (2.1)	1 (3.7)	0.794
Chylous leakage	1 (2.5)	0	0	0.240
Intraabdominal abscess	0	1 (2.1)	0	0.881
Delayed gastric emptying	1 (2.5)	6 (12.5)	4 (14.8)	0.076
Intestinal obstruction	0	0	1 (3.7)	0.140
Surgical Mortality	0	0	0	1.000

BMI: body mass index; LN: lymph node; comorbidities including cardiovascular disease, cerebrovascular accident, endocrine disease, pulmonary disease, liver cirrhosis, benign prostate hyperplasia, etc.

Some patients had more than one complication

Data were presented as mean ± SD or n (%)

Relative to patients in groups 2 and 3, patients in group 1 were more likely to receive total gastrectomy (25% vs. 6.3% vs. 3.7% for groups 1, 2 and 3, respectively, *P* = 0.005), had more retrieved lymph nodes (35.0 ± 10.4 vs. 29.7 ± 12.0 vs. 26.0 ± 10.1 for groups 1, 2 and 3, respectively, *P* = 0.005), exhibited fewer comorbidities (*P* < 0.001) and experienced longer operative time (344.9 ± 142.9 vs. 270.3 ± 109.9 vs. 311.5 ± 106.6 min for groups 1, 2 and 3, respectively, *P* = 0.019) compared to group 2 and group 3 patients. There were no differences among different age groups regarding postoperative hospital stay (9.5 ± 6.4 vs. 12.2 ± 14.0 vs. 10.7 ± 8.6 days, *P* = 0.499) and surgical complications (7.5% vs. 16.7% vs. 22.2%, *P* = 0.088).

According to an intention to treat method, one 55 y/o female patient in the MIS group was converted to open surgery. The reason for open conversion is a firm and fixed enlarged lymph node over the splenic hilum involving the pancreatic tail. Radical total gastrectomy with splenectomy, distal pancreatectomy and D2 lymphadenectomy was performed. She recovered well and was discharged 10 days after surgery.

### Open surgery

As indicated in Table 3, 244 patients received open surgery including 170 females (70%) and 73 males (30%), the median age of these patients was 68 years (range, 24–94 years). Patients were classified into three groups according to their ages. Group 1 included 72 patients (29.5%) younger than 60 years of age (47 males and 25 females), with a median age of 52 years (range, 24–59 years). Group 2 included 119 patients (48.8%) of 60 to 79 years of age (82 males and 36 females), with a median age of 70 years (range, 60–79 years). Group 3 included 53 patients (21.7%) of at least 80 years of age (41 males and 12 females), with a median age of 84 years (range, 80–94 years).

**Table 3:** Comparison of the clinicopathological differences and operative outcomes of open gastrectomy according to age

	<60 yr	60–79 yr	≧80 yr	*P* value
	*n* = 72	*n* = 119	*n* = 53	
Gender (M/F)	47/25	82/36	41/12	0.153
Tumor size (cm)	5.4 ± 2.8	6.8 ± 18.5	4.3 ± 2.5	0.471
BMI (kg/m^2^)	23.6 ± 4.0	23.3 ± 4.0	22.6 ± 3.5	0.342
Resection extent				0.582
Subtotal/total gastrectomy	48/24	87/32	39/14	
Reconstruction method				0.185
Billroth-I	4 (5.6)	17 (14.3)	7 (13.2)	
Billroth-II	1 (1.4)	0	0	
Roux-en-Y or uncut R-Y	67 (93)	102 (85.7)	46 (86.8)	
Extent of lymphadenectomy				0.452
<D2/D2	5/64	5/109	6/47	
Retrieved LN number	36.3 ± 13.9	32.7 ± 13.0	28.2 ± 13.4	0.004
Pathological T category				0.114
T1/T2/T3/T4	15/11/30/16	28/12/45/34	17/12/17/7	
Pathological N category				0.003
N0/N1/N2/N3	22/17/16/17	43/10/26/40	29/4/12/8	
Pathological TNM stage				0.004
I/II/III	19/20/33	31/15/73	22/13/18	
Number of comorbidities				<0.001
0	46 (63.9)	22 (18.5)	9 (17)	
1	19 (26.4)	57 (47.9)	22 (41.5)	
≧2	7 (9.7)	40 (33.6)	22 (41.5)	
Operative outcomes				
Operative time	328.9 ± 101.1	312.0 ± 96.0	312.2 ± 101.2	0.475
Operative blood loss	294.1 ± 232.5	384.3 ± 457.7	366.9 ± 339.6	0.267
Postoperative hospital stay	10.8 ± 5.6	14.1 ± 12.8	15.2 ± 9.7	0.041
Surgical complications	3 (4.2)	10 (8.4)	6 (11.3)	0.317
Anastomosis leakage	2 (2.8)	2 (1.7)	1 (1.9)	0.699
Chylous leakage	0	2 (1.7)	0	0.877
Delayed gastric emptying	0	1 (0.8)	2 (3.8)	0.069
Intestinal obstruction	0	1 (0.8)	0	0.913
Wound infection	0	0	2 (3.8)	0.032
Intraabdominal abscess	0	4 (3.4)	0	0.826
Choledochofistula	0	1 (0.8)	0	0.913
Pancreatic fistula	1 (1.4)	0	0	0.195
Pneumonia	0	0	2 (3.8)	0.032
Surgical Mortality	0	1 (0.8)	1 (1.9)	0.250

BMI: body mass index; LN: lymph node; comorbidities including cardiovascular disease, cerebrovascular accident, endocrine disease, pulmonary disease, liver cirrhosis, benign prostate hyperplasia, etc.

Some patients had more than one complication

Data were presented as mean ± SD or n (%)

Relative to patients in groups 1 and 2, patients in group 3 had fewer retrieved lymph nodes (36.3 ± 13.9 vs. 32.7 ± 13.0 vs. 28.2 ± 13.4 for groups 1, 2 and 3, respectively, *P* = 0.004), were less advanced with respect to pathological N category (*P* = 0.003) and TNM stage (*P* = 0.004), suffered from more comorbidities (*P* = 0.001), and experienced longer hospital stays (10.8 ± 5.6 vs. 14.1 ± 12.8 vs. 15.2 ± 9.7 for groups 1, 2 and 3, respectively, *P* = 0.041), a higher rate of wound infection (0% vs. 0%, vs. 3.8% for groups 1, 2 and 3, respectively, *P* = 0.032) and a higher incidence of pneumonia (0% vs. 0% vs. 3.8% for groups 1, 2 and 3, respectively, *P* = 0.032).

## Discussion

In the present study, among octogenarian and older gastric cancer patients, a comparison of MIS and open surgery demonstrated that MIS was associated with less operative blood loss and a shorter postoperative hospital stay; however, there were no significant differences between MIS and open surgery with respect to the rates of surgical complications or mortality.

Our results indicated that following open surgery for gastric cancer, longer postoperative hospital stays, higher rates of wound infection rate and a higher incidence of pneumonia were observed among octogenarian and older patients than among younger patients. However, for gastric cancer patients treated using MIS, no differences were observed among different age groups with respect to postoperative hospital stay and surgical complications. Among aged patients, the use of MIS instead of open surgery might provide the benefits of fewer surgical complications and shorter postoperative hospital stays. One encouraging finding of this study is that octogenarian and older patients could recover from MIS as rapidly as younger patients.

A previous report [6] addressing the surgical outcomes of open surgery for gastric cancer indicated that even patients with early gastric cancer, older patients exhibited significantly worse overall survival rates than younger patients after curative surgery. In a series examined by Kwon et al. [4], among patients who were at least 80 years of age, MIS and open surgery produced comparable rates of 5-year overall survival and disease-free survival. As the global population ages, increasing numbers of octogenarian and older gastric cancer patients will require surgical treatment. Oncological outcomes would not be the primary concern for octogenarian and older patients after surgery. To achieve more rapid postoperative recoveries, the indications for MIS for octogenarian and older patients should be extended to include patients with advanced gastric cancer.

Although aged patients are considered to be at high surgical risk and have limited functional reserve, our results demonstrated that MIS is beneficial for octogenarian and older patients. For these patients, the use of MIS instead of open surgery was also associated with earlier recovery and no increase in surgical complications. Our future study will compare the long-term survival rates for open surgery and MIS among aged patients.

Our study had certain limitations. Because this investigation is a retrospective study, selection bias may exist. Besides, the surgeon is still a major part of choosing the surgical approach for each patient, and the patient would hardly make this decision on his own. Hence, the surgeon’s selection strongly biases the results. In particular, patients who received MIS tended to be diagnosed at an earlier stage than patients who received open surgery. Furthermore, octogenarian and older patients who underwent surgery were screened for cardiopulmonary function prior to surgery; as a result, octogenarian and older patients with poor cardiopulmonary function were excluded as candidates for surgery.

Surgeons will undoubtedly select for patients in good overall condition, particularly when screening octogenarian and older patients. However, our results indicate that for selected octogenarian and older patients, MIS produced faster postoperative recovery than open surgery. To date, few reports regarding MIS for octogenarian and older patients have been published; thus only a limited number of cases involving these patients have been examined. A future meta-analysis involving a large number of octogenarian and older patients is necessary to compare the operative outcomes of open surgery and MIS for these patients.

## Conclusion

In conclusion, MIS is an advantageous technique for octogenarian and older gastric cancer patients. Among these patients, relative to open surgery, MIS produced better postoperative recovery and comparable rates of surgical complications and mortality. MIS for gastric cancer can be performed safely in octogenarian and older patients and may be regarded as the preferred surgical approach for such patients.

## Abbreviation

MIS: Minimally invasive surgery
